# Targeting the D93 cryptic collagen epitope alters integrin α2β1-dependent cellular migration and collagen remodeling in metastatic breast cancer

**DOI:** 10.21203/rs.3.rs-8406370/v1

**Published:** 2026-01-16

**Authors:** Jordan N. Miner, Christopher L. Emmerling, Joshua D. Hamilton, Joseph Raite, Zoe Vittum, Peter C. Brooks, Andre Khalil, Karissa Tilbury

**Affiliations:** University of Maine; University of Maine; University of Maine; University of Maine; University of Maine; MaineHealth Institute for Research; University of Maine; University of Maine

**Keywords:** Breast cancer, spheroids, migration, integrin, Second Harmonic Generation (SHG) microscopy, collagen

## Abstract

Targeting the tumor-associated extracellular matrix (ECM) offers a promising strategy for breast cancer therapy. During cancer progression, collagen remodeling within the ECM exposes cryptic collagen epitope sites that antibodies can selectively recognize. Here, we investigate the therapeutic potential of targeting the D93 cryptic collagen epitope in 3D human metastatic breast cancer spheroids derived from MDA-MB-231 and MCF10CA1a (M4) cell lines embedded in collagen type I hydrogels. Treatment with monoclonal antibody (mAb) D93 reduced cellular migration into collagen type I hydrogels, an effect likely mediated by integrin α2β1. Two-photon microscopy further revealed that breast cancer cells drive the exposure of D93 sites and alter collagen architecture at both the fiber and fibril levels. Interestingly, collagen remodeling was altered more in the MDA-MB-231 spheroid models whereas the reduction in cellular migration was more pronounced in the M4 spheroid models, indicating a cell-line specific response to mAb D93. Together, these findings suggest that mAb D93 may inhibit integrin α2β1-dependent metastatic migration in breast cancer.

## Introduction

Breast cancer is a heterogeneous disease and thus advancements in precision medicine is essential for optimal patient treatment.^1^ This is especially true for triple negative breast cancer (TNBC) which is negative for the estrogen receptor (ER), the progesterone receptor (PR) and human epidermal growth factor 2 (HER2).^1-3^ Due to the lack of these receptors, TNBC is typically treated with chemotherapy and has a poor clinical prognosis.^3^ While some treatments target cells directly, others target the extracellular matrix (ECM) surrounding the tumor.^1,4^ The ECM, composed primarily of collagen, plays a critical role in cancer growth and metastasis.^4^ Collagen is a triple helical protein that not only provides mechanical support,^5^ but also mediates signaling which impacts cellular behaviors including adhesion, proliferation, and migration.^4^ During cancer progression, collagen is remodeled to expose cryptic collagen epitope sites.^4^ One such site, HU177, has been shown to influence cellular migration in cancers such as melanoma^6^ and ovarian cancer.^7^ Additionally, the monoclonal antibody (mAb) targeting the HU177 site selectively binds to denatured collagen.^4,8,9^ The humanized version of mAb HU177, termed mAb D93, underwent a phase-I human clinical trial in advanced refractory cancer patients and produced encouraging results.^4,10^ The treatment was well-tolerated without dose-limiting toxicities and the single breast cancer patient had disease stabilization for approximately 1–2 months.^4,10^ Building on these findings, we investigated the therapeutic potential of the D93 site in breast cancer using *in-vitro* human spheroid models, which provide a more physiologically relevant alternative to traditional 2D-tissue cultures.^11,12^

Cells interact with cryptic collagen epitope sites via integrins which mediate cell-to-cell and cell-to-ECM adhesion and play vital roles in cell signaling cascades.^13-15^ Four integrins directly bind to collagen: α1β1, α2β1, α10β1, and α11β1.^14^ A recent study demonstrated that collagen-binding integrins α2β1 and α10β1 can bind the HU177 epitope site in ovarian cancer.^7^ Integrins play a part in remodeling the ECM,^13,16^ leading to the formation of tumor associated collagen signatures (TACS).^17,18^ TACS3, characterized by linearized collagen perpendicular to the tumor boundary, is clinically linked to poor patient prognosis.^17,18^ These collagen signatures can be imaged using label-free Second Harmonic Generation (SHG) microscopy.^18,19^ SHG is a coherent process where the SHG signal is dependent on the quasi-phase-matching of the collagen structure.^20^ By simultaneously measuring both the forward and backward-generated SHG signals, the Forward/Backward (F/B) Ratio provides insight into collagen fibril packing.^20-25^ Previous work showed a decrease in the F/B Ratio during progression from Ductal Carcinoma *In Situ* (DCIS) to Invasive Ductal Carcinoma (IDC).^21^ Measurements at the tumor-stroma interface can predict metastasis-free survival in IDC patients.^22^ In *in-vitro* collagen gels, cellular migration has previously been correlated with the F/B Ratio and local collagen microstructure changes were observed near cellular boundaries.^26^

Given prior evidence that D93 binding sites are exposed in MDA-MB-231 mice xenografts,^27^ we hypothesized that mAb D93 could be a potential therapeutic option for breast cancer patients. To test this, we developed 3D spheroid models of two immortalized breast cancer cell lines – MDA-MB-231 and MCF-10CA1a^28-30^ (M4) – embedded in collagen type I (COL1) hydrogels. The M4 cell line was derived from the non-tumorigenic MCF10A cell line through transfection with constitutively active *HRAS* (yielding the MCF10AT1 cell line) followed by serial passages in immunodeficient mice to confer a highly metastatic phenotype.^28-30^ Thus, we investigated the effects of mAb D93 on cellular migration and collagen remodeling. To assess the role of collagen-binding integrin α2β1 in this process, we generated α2β1 knockdown cell lines using shRNA transduction. Finally, we quantified the impact of mAb D93 treatment on cellular migration in both control and α2β1 knockdown spheroids and analyzed collagen architecture at the spheroid boundaries, migratory cell front, and distant cell-free regions.

## Results

### Addition of mAb D93 reduces cellular migration.

**Figure 1.**
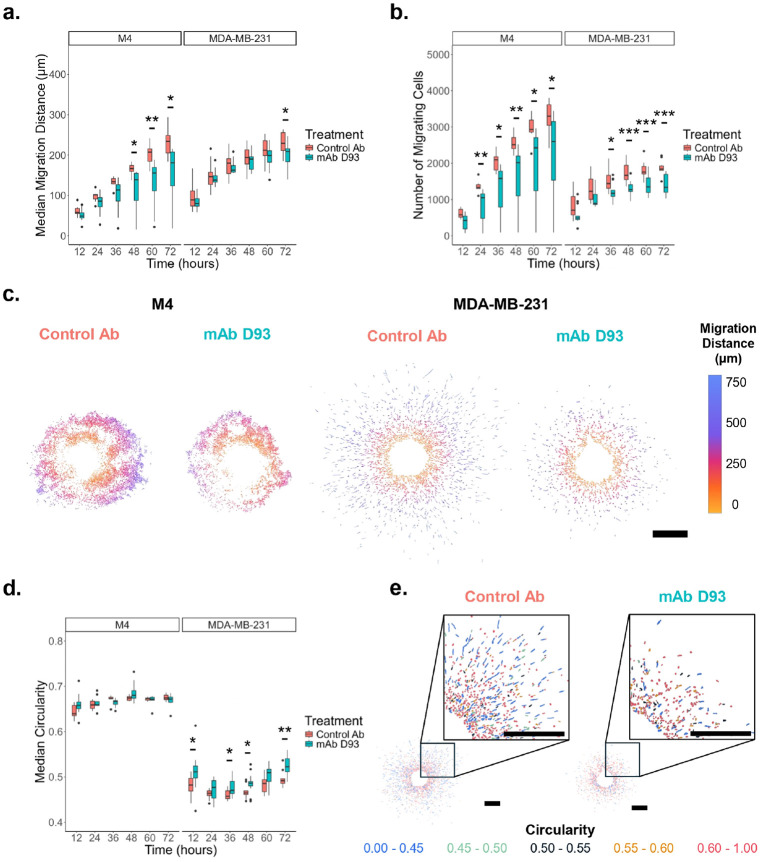
Treatment with the mAb D93 Reduces Cellular Migration into Collagen Type I. Reduction of (a) median migration distance and (b) number of migrating cells when treated with mAb D93 compared to the Control Ab is present in both cell lines but is more pronounced in the M4 cell line. M4: N = 8 for mAb D93 and N = 7 for Control Ab. MDA-MB-231: N = 13 for mAb D93 and N = 10 for Control Ab. (c) 72-hour masks of migrating cells color coded by distance from the spheroid boundary of models. Scale bar = 500 μm. (d) Morphological differences were evaluated using the median circularity (defined as 4π*area/perimeter^2^) of all cells per spheroid model where 1 is a perfect circle and 0 is a perfect line. A significant decrease in elongation can be seen with the mAb D93 compared the Control Ab for the MDA-MB-231 cell line. The M4 cell line had limited morphological differences. M4: N = 8 for mAb D93 and N = 7 for Control Ab. MDA-MB-231: N = 13 for mAb D93 and N = 10 for Control Ab. (e) Representative images of MDA-MB-231 spheroids at 72 hours where each individual cell is color coded based off its circularity value. Visibly more blue-colored (elongated) cells can be seen in the Control Ab condition. Scale bars = 500 μm.

MDA-MB-231 and MCF-10CA1a (M4) spheroids were embedded into 2 mg/mL collagen type I hydrogels and treated with either mAb D93 or a control antibody (IgG1) for 72 hours. Treatment with mAb D93 significantly reduced both the median migration distance (Fig. 1a) and number of migrating cells (Fig. 1b) in both the M4 and MDA-MB-231 cell lines. The effect of mAb D93 was more pronounced in M4 spheroids, which exhibited larger differences between treatment and control groups compared to MDA-MB-231 spheroids. Different migration patterns were visually observed; M4 cells appeared to migrate more collectively whereas MDA-MB-231 cells appeared to migrate more individually (Fig. 1c). In the MDA-MB-231 cell line, morphology was treatment-dependent; cells exposed to mAb D93 were more circular, while control antibody treated cells had a more aggressive, elongated morphology (Fig. 1d,e). In contrast, the M4 cells maintained higher median circularity values compared to MDA-MB-231 cells. Furthermore, M4 cellular morphology was not impacted by mAb D93 despite the reduction in median migration distance and number of migrating cells (Fig. 1d).

### Cellular migration reduction is driven by integrin α2β1.

**Figure 2.**
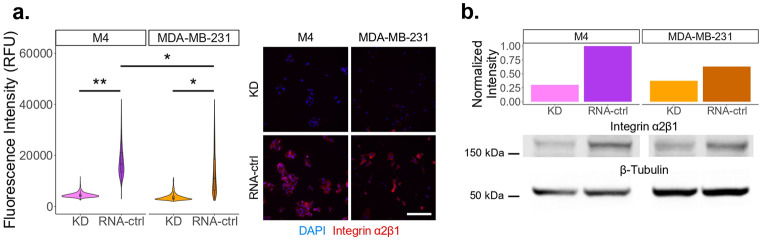
Validation of the integrin α2β1 KD cell lines. The KD populations were validated using (a) fluorescence labeling and (b) Western blotting. The M4 RNA-ctrl cell line had higher integrin α2β1 expression compared to the MDA-MB-231 RNA-ctrl cell line. N = 3 per cell line for fluorescence statistical analysis. Scale bar = 200 μm. β-tubulin was used as the loading control for the Western blot. The uncropped blot can be seen in **Supplementary Figure 2.**

Previous research has shown that both integrin α2β1 and α10β1 can bind to the D93 collagen epitope.^7^ Furthermore, integrin α2β1 has been shown to play a role in cancer progression.^31-34^ Thus, integrin α2β1 knockdown (KD) cell lines were generated, validated, and maintained with at least 60% KD to identify the role of α2β1 integrin in binding the cryptic D93 collagen epitope and in cellular migration (Fig. 2; **Supplementary Figs. 1,2**). Moreover, M4 was shown to have higher baseline integrin α2β1 compared to the MDA-MB-231 cell line (Fig. 2). To verify integrin α2β1 KD did not alter the expression of other collagen-binding integrins (α1β1, α10β1, and α11β1) or the fibronectin-binding integrin αVβ1, fluorescent labeling was performed where no statistical differences were observed between the KD and control (RNA-ctrl) cell lines (**Supplementary Fig. 3**).

**Figure 3.**
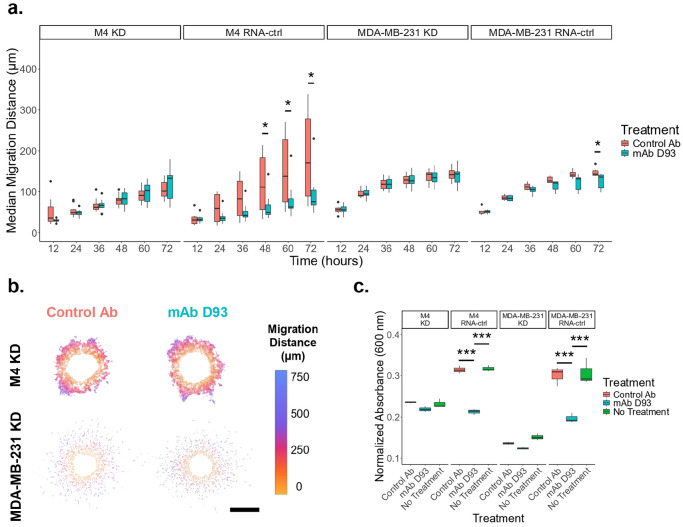
Impact on cellular migration is most likely driven by integrin α2β1. (a) There is no statistical significance in the median migration distance between the mAb D93 and the Control Ab treatments in the M4 KD and MDA-MB-231 KD cell lines. Significant differences are seen in the M4 RNA-ctrl and MDA-MB-231 RNA-ctrl cell lines at later time points, comparable to the wildtype migration. M4 KD: N = 10 for mAb D93 and N = 10 for Control Ab. M4 RNA-ctrl: N = 10 for mAb D93 and N = 10 for Control Ab. MDA-MB-231 KD: N = 14 for mAb D93 and N = 7 for Control Ab. MDA-MB-231 RNA-ctrl: N = 6 for mAb D93 and N = 7 for Control Ab. (b) 72-hour masks of migrating cells color coded by distance from the spheroid boundary of models treated with (first column) Control Ab and (second column) mAb D93. Scale bar = 500 μm. (c) mAb D93 (at 100 μg/mL) significantly reduces the adhesion of both the M4 RNA-ctrl and MDA-MB-231 RNA-ctrl cell lines whereas there are no statistical differences in the M4 KD and MDA-MB-231 KD cell lines. M4: 7 minutes. MDA-MB-231: 9 minutes. N = 3 for all groups.

Using these validated KD lines, we repeated the 3D migration assays. The KD spheroids showed no significant differences between mAb D93 and control antibody treatments, indicating that the reduction in cell migration observed in the wild-type cells was mediated, at least in part by integrin α2β1 (Fig. 3a,b). Integrin α2β1 KD was sustained throughout migratory studies and no observable differences in the expression of integrin α10β1 expression levels were observed in 3D culture (**Supplementary Fig. 4**). The RNA-ctrl cell lines maintained similar migration characteristics to the wild-type cell lines (Figs. 1a & 3a). A 2D adhesion assay with plates coated at 5 μg/mL of denatured collagen type I demonstrated that the mAb D93 treatment significantly reduced adhesion in the M4 and MDA-MB-231 RNA-ctrl cell lines, but not in the KD cell lines (Fig. 3c). Furthermore, the KD cell lines had decreased adhesion compared to their RNA-ctrl counterparts. Together, these results corroborate that mAb D93 was primarily blocking the ability of integrin α2β1 to bind to denatured collagen in both breast cancer cell lines.

### D93 binding sites are exposed.

**Figure 4.**
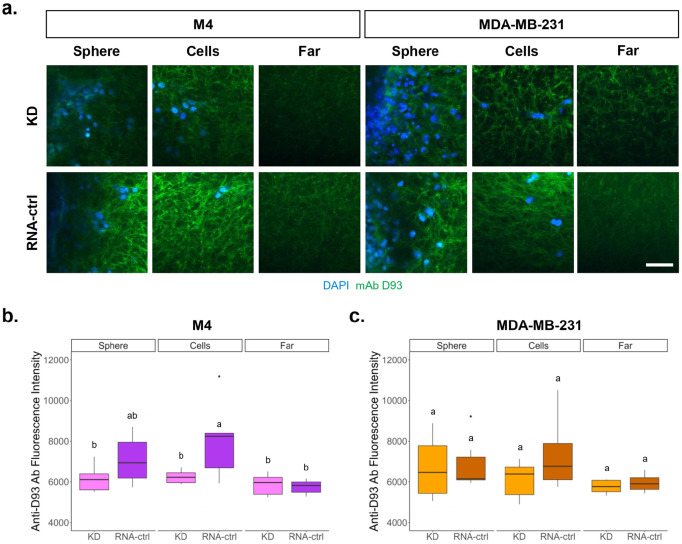
Cells are exposing the D93 sites. (a) After 72 hours, the models were fixed with 4% PFA and labeled with 20 μg/mL goat anti-human IgG Alexa Fluor Plus 488 and 1 μg/mL DAPI. Samples were transferred to an upright microscope compatible 3D printed holder and two-photon images were acquired at 40X of the anti-D93 antibody (green) and nuclei (blue). Scale bar = 50 μm. (b) Quantitative analysis revealed a statistically significant increase in anti-D93 antibody intensity the M4 RNA-ctrl cell line near vs far away from the spheroid, (c) The MDA-MB-231 RNA-ctrl cell line had a non-significant increase near vs far away from the spheroid boundary (distinct groups indicated by the letters above the boxplots where groups that do not share the same letter are significantly different). N = 6 for all groups.

To understand if the cells were exposing the D93 sites, the 72-hour migration spheroid models were fixed and both the cryptic collagen D93 sites and the cell nuclei were labeled for two-photon fluorescence microscopy studies. Two-photon fluorescence images were captured at three regions of the model: (1) spheroid boundaries (sphere), (2) migratory cell front (cells), and (3) distant cell-free regions (far). In RNA-ctrl models, the intensity of mAb D93 staining was trending higher in the MDA-MB-231 cell line and statistically higher in the M4 cell line in the cells region compared to the far region (Fig. 4). This indicates that the cells themselves are remodeling collagen to expose the D93 sites, and they are not fully exposed during collagen gel formation. High variability in D93 staining intensity was seen in the sphere and far regions of both RNA-ctrl cell lines whereas the far regions and M4 KD models had lower variability. Furthermore, in the KD cells, the fluorescent intensity of the mAb D93 was consistent throughout the sphere, cells, and far regions, potentially indicating that cells with reduced α2β1 expression have reduced capacity to remodel and expose the cryptic D93 collagen binding sites.

### Collagen fibril packing is altered.

**Figure 5.**
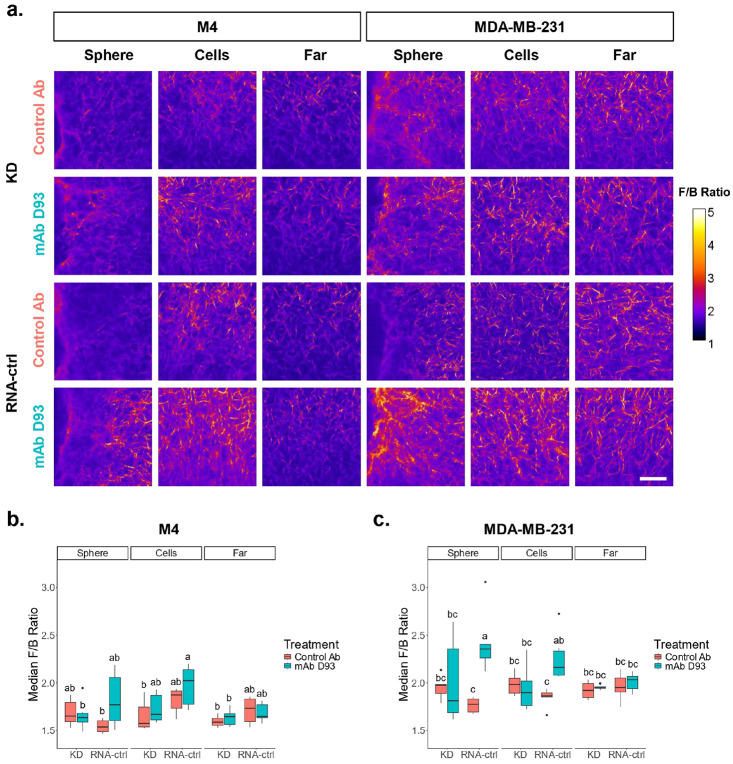
Cells are remodeling their local microenvironment. (a) Representative F/B Ratio images at all three regions of interest. In the spheroid boundary images (sphere), the spheroid is positioned on the left. Scale bar = 50 μm. Analysis of the F/B Ratio in the (b) M4 and (c) MDA-MB-231 cell lines. There were no statistical differences in the M4 RNA-ctrl group between treatments whereas in the MDA-MB-231 RNA-ctrl group treatment with the mAb D93 increased the median F/B Ratio in the sphere and cells region. No statistical differences were detected in the far regions in all cell lines (distinct groups indicated by the letters above the boxplots where groups that do not share the same letter are significantly different). N = 6 for all group.

After completion of 72-hour migration experiments, SHG microscopy was performed on fixed, fluorescently labeled (mAb D93 and cell nuclei) models to evaluate collagen organization. Collagen fibril packing was quantified using the ratio of forward to backward propagating SHG signal (F/B Ratio), which is based on quasi-phase matching and infers collagen fibril diameters and packing assemblies.^20,35^ In MDA-MB-231 RNA-ctrl spheroids treated with mAb D93, the F/B Ratio was significantly elevated at both the spheroid boundary and within the migratory cell regions compared to the control antibody treated spheroids (Fig. 5a,c). In contrast, the M4 RNA-ctrl spheroids showed only modest, non-significant increases in the F/B Ratio at both the sphere and cells regions after mAb D93 treatment (Fig. 5a,b). In both cell lines, the spheroid-collagen boundary demonstrated the greatest differences in the F/B Ratio and physical collagen alterations. In the MDA-MB-231 RNA-ctrl spheroids, mAb D93 treatment produced dense, localized collagen signatures; whereas control antibody treated spheroids exhibited more diffuse collagen distribution (Fig. 5a). By comparison, M4 RNA-ctrl spheroids showed stronger diffuse collagen signatures in both the mAb D93 and control antibody treatments. This is consistent with the observed collective migratory behavior of M4 cells, in contrast to the observed single-cell migration of MDA-MB-231 cells (Fig. 1c).

Meanwhile, integrin α2β1 KD models of both cell lines showed no significant changes in the F/B Ratios across treatment groups (Fig. 5). This could be in part due to less cells migrating into the collagen gel (Figs. 1a, 3a). Notably, high variability in the F/B Ratio was observed at the spheroid boundary of mAb D93 treated MDA-MB-231 KD spheroids (Fig. 5c). In both cell lines, the F/B Ratios in far regions remained unchanged between the RNA-ctrl and the KD spheroid models regardless of treatment, indicating that the alterations to the F/B Ratio are associated with localized cellular remodeling in the presence or absence of mAb D93 antibodies. Taken together, these observations may suggest that once cells migrate into the collagen gel, the binding of mAb D93 may facilitate the formation of more complex remodeled fibril assemblies associated with an increased F/B Ratio.

**Figure 6.**
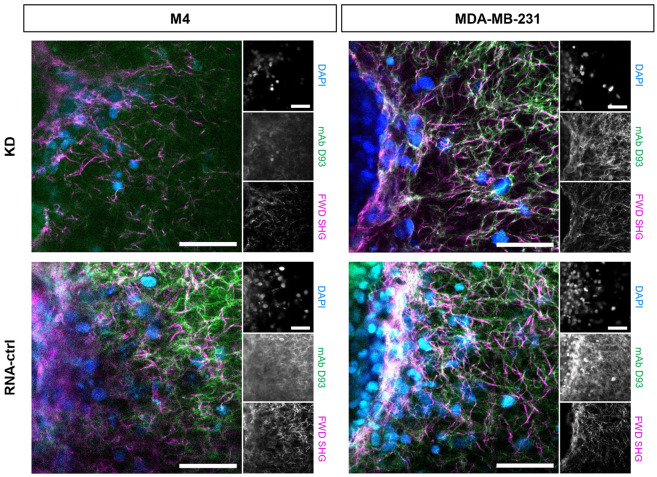
Anti-D93 antibody is not always localized with the forward SHG signal. Representative images of the localization of the anti-D93 antibody (green) and the forward SHG signal (magenta) at the spheroid boundary. Cells are labeled with DAPI (blue). The SHG signal has high overlap with the anti-D93 antibody. However, the anti-D93 antibody has many regions not localized with the SHG signal. Scale bars = 50 μm.

To further investigate the spheroid boundary differences, we examined the localization of mAb D93 relative to the forward SHG signal. In the MDA-MB-231 spheroids, there was a high degree of overlap between SHG and mAb D93 signals in both the RNA-ctrl and α2β1 integrin KD models (Fig. 6). In contrast, the M4 spheroid models showed multiple areas of positive mAb D93 signal without forward SHG signal, potentially reflecting collagen degradation too extensive to generate SHG signal.

### Collagen fiber alignment is altered.

**Figure 7.**
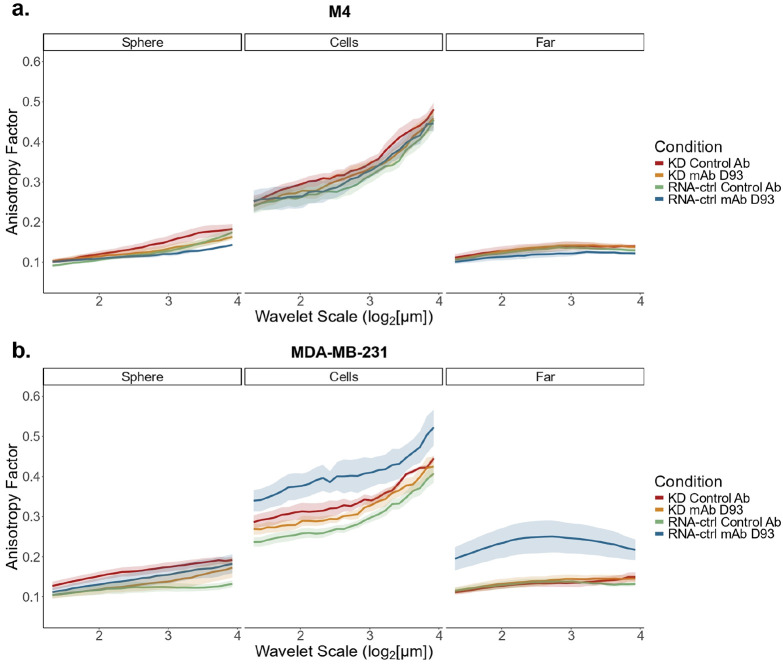
Collagen organization is altered when treated with mAb D93 in MDA-MB-231 RNA-ctrl cells. Forward SHG images from 72-hour migration models were evaluated for collagen organizational differences using the 2D WTMM anisotropy method over multiple size scales in (a) M4 and (b) MDA-MB-231 cell lines. The solid line represents the mean while the shaded region is the standard error. See Supplementary Figure 5 for statistics. N = 6 for all groups.

Collagen fiber alignment has been correlated with poor patient prognosis.^18^ To quantify collagen alignment, we applied the 2D wavelet transform modulus maxima (WTMM) anisotropy method, where lower anisotropy values indicate isotropic (disordered) and higher values indicate anisotropic (aligned) fibers.^36,37^ The 2D WTMM anisotropy method is a multiscale approach where the continuous wavelet transform acts like a mathematical microscope to characterize and quantify directionality of the SHG image gradient over a continuous range of size scales. In both cell lines, most conditions exhibited a scale-dependent increase in anisotropy factor; however, the magnitude of this increase was comparable across conditions (Fig. 7; **Supplementary Fig. 5**). Interestingly, the M4 cell line had minimal statistical differences in collagen alignment detected between treatment groups for both the KD and RNA-ctrl spheroids (Fig. 7a; **Supplementary Fig. 5a**). In contrast, MDA-MB-231 RNA-ctrl spheroids treated with mAb D93 showed an increase in collagen alignment compared to the control antibody condition in both the cells and far regions (Fig. 7b; **Supplementary Fig. 5b**). The substantial increase in alignment observed in the far region was especially notable, as this area showed no differences in the F/B Ratio and no cells were present to mediate localized remodeling.

To further investigate this effect in the far region, we employed CT-FIRE to quantify single collagen fiber properties rather than the bulk field of view alignment quantified by the 2D WTMM anisotropy method (**Supplementary Fig. 6**).^38^ CT-FIRE analysis corroborated the 2D WTMM anisotropy method results, with mAb D93 treated MDA-MB-231 RNA-ctrl spheroids having significantly altered median fiber angle compared to the control treated group. Furthermore, in this same region, an increase in median fiber length and width were also observed when treated with mAb D93. These changes were not observed in the M4 RNA-ctrl cell line. Interestingly, median fiber width was significantly higher in the M4 KD compared to the M4 RNA-ctrl models regardless of treatment. In both analysis methods, the most pronounced collagen remodeling was observed in MDA-MB-231 RNA-ctrl spheroids treated with mAb D93, suggesting that mAb D93 promotes alignment of collagen fibers, whereas M4 spheroids primarily exhibited collagen degradation lacking organization.

## Discussion

This study highlights the utility of metastatic breast cancer spheroids embedded in a 3D microenvironment as a model for characterizing 3D cellular migration and collagen remodeling. Previous work has identified the D93 cryptic collagen epitope in both MDA-MB-231 mouse xenografts and human breast tissue samples.^27^ Building on this foundation, and motivated by promising outcomes of a phase I clinical trial evaluating mAb D93,^4,10^ we investigated how targeting the cryptic D93 collagen site influences cellular behavior and collagen remodeling in two metastatic breast cancer cell lines, MDA-MB-231and MCF-10CA1a (M4).^28,29^

Treatment of 3D embedded spheroids with mAb D93 significantly reduced cellular migration in both models with a stronger effect observed in M4 cells (Fig. 1a,b). In addition, mAb D93 treatment altered the morphology of migrating MDA-MB-231 cells; increasing their circularity values which are associated with a less aggressive phenotype (Fig. 1d,e). In contrast, M4 migrating cells did not alter their cellular morphology consistent with their baseline circular phenotype in 2D culture (Fig. 1d). These findings suggest that inherent cellular morphology may shape both migratory capacity and responsiveness to D93 antibody treatment.

Given the ability of mAb D93 to reduce cellular migration, we next sought to determine which collagen-binding integrin’s ability to bind to denatured collagen was being blocked by mAb D93. Previous studies have shown that both α2β1 and α10β1integrins can interact with the D93 collagen epitope.^7^ Since both cell lines expressed elevated levels of integrin α2β1, we generated and validated integrin α2β1 knockdown (KD) cell lines (Fig. 2; **Supplementary Figs. 1–4**). When KD spheroids were embedded in collagen type I hydrogels, they showed no difference in migration between treatment groups (Fig. 3a,b). These findings indicate that mAb D93 primarily acts by preventing integrin α2β1 from binding to the cryptic D93 site. Furthermore, no reduction in cellular adhesion was observed in the KD cell lines when treated with mAb D93 (Fig. 3c). Importantly, the expression level of other collagen-binding integrins were not altered in the integrin α2β1 KD lines (**Supplementary Fig. 3**), supporting that integrin α2β1 is a critical mediator of cellular migration and adhesion in both cell lines. Nonetheless, alternative mechanisms can contribute to migration into collagen hydrogels including secretion of matrix metalloproteinases (MMPs),^39,40^ lysyl oxidase (LOX),^41^ and cytokines^42^ which were not explored in this study. Interestingly, the MDA-MB-231 cells continued to have single-cell migration phenotype, whereas the M4 cells continued to migrate more collectively, regardless of integrin α2β1 expression (Fig. 1c; Fig. 3b). These findings suggest that while integrin α2β1 is essential for migration efficiency, additional pathways regulate the mode of migration. Future studies dissecting these alternative mechanisms may provide new opportunities to selectively target single cell versus collective migration patterns, thereby refining therapeutic strategies for different breast cancers.

D93 site exposure was evaluated using two-photon fluorescence microscopy, focusing on three regions of interest in the 3D model: (1) spheroid boundaries (sphere), (2) migratory cell front (cells), and (3) distant cell-free regions (far). In M4 RNA-ctrl cell lines, mAb D93 binding was significantly higher near the spheroid compared to distant regions (Fig. 4). In contrast, MDA-MB-231 RNA-ctrl spheroids showed no statistically significant differences between regions, although antibody binding near the spheroid trended higher than in more distant areas. Together with the reduced mAb D93 signal observed in the α2β1 integrin KD models, these findings suggest that M4 cells locally remodel their environment to expose D93 sites, at least in part through α2β1 integrin activity.

Second Harmonic Generation (SHG) microscopy revealed differences in collagen architecture at both the fibril and fiber scale. In MDA-MB-231 RNA-ctrl models, treatment with mAb D93 significantly increased the F/B Ratio in the sphere and cells regions compared to control antibody treatment, but not in the far region (Fig. 5a,c). The M4 RNA-ctrl models showed a similar trend; though they did not reach statistical significance (Fig. 5a,b). Previous studies in human breast tissue have shown that healthy breast exhibits a higher F/B Ratio than invasive ductal carcinoma,^21^ suggesting that mAb D93 treatment may partially restore “normal” collagen fibril packing.

Additionally, in MDA-MB-231 RNA-ctrl models, there was increased collagen fiber anisotropy (collagen fiber alignment) in both the cells region, and, interestingly, the far region when treated with mAb D93 at all size scales; whereas M4 cells showed no major differences (Fig. 7; **Supplementary Fig. 5**). Quantification of individual fiber characteristics in the far region using CT-FIRE revealed increased fiber length and width in the MDA-MB-231 RNA-ctrl spheroid treated with mAb D93 (**Supplementary Fig. 6g,h**). We hypothesize that the lack of detectable alignment differences in the sphere region is due to the spheroid boundary dominating the anisotropy calculations. In integrin α2β1 KD models, differences in F/B Ratio and anisotropy between treatment groups were minimal (Figs. 5 & 7; **Supplementary Fig. 5**) supporting that integrin α2β1-dependent mechanisms drive collagen remodeling, which in turn contribute to altered cellular behaviors.

Taken together, treatment with mAb D93 effectively reduces integrin α2β1-mediated cellular migration, although the mechanisms of D93 site exposure appear to differ between the two cell lines. In MDA-MB-231 RNA-ctrl spheroids, mAb D93 treatment induced a sizeable increase in collagen surrounding the spheroid-collagen region (Figs. 5a & 6) and enhanced collagen alignment in both the cells and far regions (Fig. 7). These observations suggest that MDA-MB-231 cells mechanically remodel collagen fibers in response to mAb D93 treatment. Since the collagen hydrogels adhere to the walls of the 96-well plate, mechanical forces generated by the cells could propagate alignment into the far regions of the hydrogel.^43,44^ This fiber straightening may also explain why mAb D93 treatment was less effective in MDA-MB-231 cells compared to M4 cells, as aligned collagen fibers could facilitate migration despite mAb D93 blocking α2β1 integrins from binding to denatured collagen.^17,18^ Notably, these features were absent in the integrin α2β1 KD models, indicating integrin α2β1 may assist in mechanical collagen remodeling.

By contrast, M4 RNA-ctrl spheroids exhibited more diffuse collagen signatures near the spheroid-collagen boundary (Figs. 5a & 6) with no changes in collagen alignment across regions (Fig. 7) and a slight, non-significant decrease in fiber length in the far region in mAb D93 treated models (**Supplementary Fig. 6c**). These results suggest that in M4 models, D93 sites are primarily exposed through collagen degradation, with integrin α2β1 playing a key role in facilitating site accessibility (Fig. 4a,b). This hypothesis is further supported by regions with positive mAb D93 signal without corresponding forward SHG (Fig. 6). Extensive collagen degradation decreases SHG signal generation due to the loss of phase-matching and may explain the relative insensitivity of the F/B Ratio in these regions (Fig. 5a,b). Future studies could employ rheometry or nano-indentation to quantify the mechanical properties of collagen hydrogels. Additionally, to study the contractile forces of these cells, collagen gel contraction studies may be used to further understand the cell-matrix interactions.

This study was limited to a single collagen concentration (2 mg/mL) and relied on mono-culture spheroids, which do not fully capture the biochemical and biomechanical complexities of the tumor microenvironment. Incorporating additional cell types, such as fibroblasts, macrophages, or using patient-derived organoids could provide a more physiologically relevant model. Spheroid formation was performed using Matrigel, which is known to exhibit batch-to-batch variability.^45^ To minimize this source of variation, all experiments utilized the same Matrigel batch. The two-photon microscopy experiments included a limited number of replicates (n = 6) and were conducted at a single time point (72-hours). Future studies could employ live-cell SHG imaging to monitor the temporal dynamics of collagen remodeling. This may particularly be interesting to explore if cell behavior and collagen remodeling are altered with the treatment of mAb D93 antibodies after cellular migration has initiated. Additionally, characterization of secreted factors such as MMPs, LOX, and cytokines could provide insight into collagen remodeling mechanisms beyond direct cell-collagen interactions. The use of inhibitors such as GM6001 would allow assessment of the specific contribution of MMP activity in exposing the cryptic D93 sites.

Overall, these findings identified the D93 collagen epitope as a key regulator of breast cancer cell migration through integrin α2β1, demonstrating that antibody blockade of this site not only suppresses migration but also influences extracellular matrix organization. By linking cryptic collagen epitopes to both tumor cell dynamics and matrix remodeling, this work provides new mechanistic insight into tumor-matrix interactions and supports further evaluation of mAb D93 as a potential therapeutic approach for limiting breast cancer progression.

## Materials and Methods

### Cell Culture Conditions.

MDA-MB-231 cells were purchased from the American Type Cell Culture Collection (ATCC). MCF10CA1a.cl1 cells (M4) were purchased from the Animal Model and Therapeutic Evaluation Core (AMTEC) Karmanos Cancer Institute, Wayne State University. Both cell lines are established human breast cancer cell lines obtained from approved repositories; their use did not require additional institutional review board approval or informed consent. MDA-MB-231 cells were cultured in DMEM/F12 supplemented with 10% fetal bovine serum and 1X penicillin-streptomycin. M4 cells were cultured in DMEM/F12 supplemented with 1.05 mM calcium chloride (500 mM stock solution), 20% horse serum, and 10 mM HEPES (1 M stock solution). Both culture mediums were passed through a 0.22 μm filter before use. Cells were maintained at 37°C and 5% CO_2_ in a cell culture incubator in T25 flasks and passaged twice a week with 1X Trypsin EDTA at splitting ratios of 1:6 – 1:10 for both cell lines.

### Spheroid Generation.

Spheroids were generated using the centrifugation method as described in Karrobi et al.^46^ Briefly, cells were detached from the T25 flasks using 1X Trypsin EDTA and resuspended at 1 x 10^6^ cells/mL in cold complete medium (cells counted using a hemocytometer). A solution of complete cell medium with 4% stock cell suspension and 2.5% Matrigel Matrix was added to a reagent reservoir. It is important to keep all solutions cold and the Matrigel on ice when generating spheroids as an increase in temperature can make the Matrigel prematurely polymerize, resulting in poor spheroid creation. 100 μL of the cell/Matrigel suspension was added using a multichannel pipette to each well of a chilled 96-well ultra-low attachment plate for an initial seeding density of 4,000 cells per spheroid. The plate was centrifuged at 1000 rpm (101 rcf) for 30 minutes at room temperature. Loose cell clumping was confirmed under a brightfield microscope before allowing spheroids to form in the incubator at 37°C and 5% CO_2_. If no clumping was seen, the plate was centrifuged for an additional 10 minutes. Spheroids reached a diameter of 350–450 μm within 2–3 days of formation.

### Spheroid Screening.

To ensure uniform spheroid selection, brightfield images were acquired using an Agilent BioTek Cytation 5 Cell Imaging Multimode Reader and further processed using a custom FIJI macro (ImageJ 1.54f). A single image at the spheroid’s center (set using beacons) was acquired using a 10X, 0.3 NA objective (16-bit TIF, 1992 x 1992 pixels, 1389 x 1389 μm, LED intensity: 10, integration time: 5 ms, camera gain: 25). The images were converted to 8-bit and pre-processed by de-speckling and using a 10-pixel gaussian blur. Then, an auto threshold was applied using the Otsu method. Solid spheroid masks were generated by using the “Fill Holes” binary operation prior to calculating the area, shape descriptors, and ellipse fitting parameters. Spheroids were selected for further experimentation if the circularity was greater than 0.55, the roundness was greater than 0.80, and if the major and minor axis of the ellipse fitting were between 350–450 μm (**Supplementary Fig. 7**).^47,48^

### Spheroid Embedding.

A 2 mg/mL collagen type I hydrogel solution (Corning Collagen I, rat tail collagen, high concentration) was prepared on ice by mixing acid-solubilized high concentration collagen type I neutralized with an equal volume of 100 mM HEPES (in 2X PBS, pH 7.3), 5 mM magnesium chloride (200 mM stock solution in 1X PBS), 0.5 mM manganese chloride (20 mM stock solution in Milli-Q water), and 1X PBS (pH 7.4). 100 μL of the collagen hydrogel solution was added to each well in a 96-well flat bottom plate. Screened spheroids were washed with 1X PBS and added with 2 μL of 1X PBS to the collagen hydrogel solution with a cut P10 pipette tip to avoid compressing the spheroid. To ensure the spheroids were fully surrounded by collagen and not contained in a PBS bubble, the spheroids were mixed thoroughly with the collagen solution using a cut P200 pipette tip. The spheroids were centered in the xy-plane by gently positioning them in the collagen hydrogel solution with a P10 pipette tip, ensuring the plate remained cold on ice. Once all spheroids were centered in the xy-plane, the plate was placed in an incubator at 37°C and 5% CO_2_ and allowed to softly polymerize for 3 minutes. The plate was then rotated every 1 minute for 3 full rotations to center the spheroids in the z-plane before fully polymerizing upright for 90 minutes. 200 μL of DMEM/F12 without phenol red was added on top of the polymerized collagen hydrogels with 20 μg/mL of either mAb D93 (acquired from Tracom Pharmaceuticals, San Diego, CA) or human IgG1 isotype control antibody.

### 3D Cellular Migration.

After spheroid embedding, the spheroid plate was added to an Agilent BioTek Cytation 5 Cell Imaging Multimode Reader with an external CO_2_ regulator. The internal temperature was set at 37°C with a 2°C gradient and the CO_2_ regulator was set at 5% to maintain a proper environment for cell culture. Widefield brightfield microscopy images were captured automatically with a 10X, 0.3 NA objective (16-bit TIF, 1992 x 1992 pixels, 1389 x 1389 μm, LED intensity: 10, integration time: 12 ms, camera gain: 24) every 12 hours for a total duration of 72 hours in a 2x2 montage (10% overlap) and a 41-image z-stack (10.1 μm step size). Using the Gen5 analysis software (version 3.12.08), a single image projection was produced per time point using the focus stacking method and images were stitched using the Linear Blend method.

### Image Analysis: Cellular Migration.

A custom FIJI macro was created to quantify cellular migration. To segment the spheroid bodies, the image projections were converted to 8-bit, Gaussian blurred with a radius of 10 pixels, and an auto threshold was applied using the Max Entropy method (MDA-MB-231) or the Minimum method (M4). The coordinates of the spheroid boundary were saved as a CSV file for use in later analysis. Migrating cell masks were generated for the MDA-MB-231 models by subtracting a median filter image (radius = 20 pixels) from the original image, enhancing local contrast (CLAHE, blocksize = 127, histogram bins = 256, maximum slope = 10), de-speckling the image, and auto thresholding using the Max Entropy method. For the M4 migrating cell masks, the background was subtracted (radius = 20 pixels), de-speckled twice, and an auto threshold was applied using the Triangle method. Any cell in the cell mask detected in the spheroid mask was removed to keep only the migrating cells. Furthermore, to prevent the detection of uneven locations in the collagen as cells, each image was manually evaluated to remove those locations. Finally, a FIJI script using the “Analyze Particles” function was developed to generate a CSV file of all migrating cells (Area > 40 pixels) with data analyzing the area, center of mass, shape descriptors, and ellipse fitting. The migrating cell CSV file was imported into RStudio (version 2024.04.2 + 764 with R version 4.4.1) and the median migration distance (the closest value in the spheroid boundary coordinates to the center of mass of each migrating cell assuming a linear migration pattern by using the RANN package version 2.6.2), median circularity, and number of migrating cells per spheroid per time were calculated. ggplot2 (version 3.5.1) was used to graph the data.

### Integrin α2β1 Knockdown.

α2β1 and Control Particles-A shRNA Lentiviral Particles were purchased from Santa Cruz Biotechnology. The manufacturer’s protocol was used with slight modifications. Prior to performing the knockdown (KD), the correct polybrene and puromycin dihydrochloride concentrations were determined for each cell line by plating 7,000 cells per well in a 96-well plate. Concentrations from 0 to 15 μg/mL in 2.5 μg/mL increments were tested. The optimal polybrene concentration was determined to be the highest concentration where no cytotoxic effects can be seen in the cells 18 hours after seeding (7.5 μg/mL for MDA-MB-231 and 5 μg/mL for M4). The optimal puromycin dihydrochloride concentration was determined to be the lowest concentration that kills all cells within 72 hours (7.5 μg/mL for MDA-MB-231 and 2.5 μg/mL for M4). To perform the KD experimentation, three wells in a 96-well plate were seeded with 7,000 MDA-MB-231 or M4 cells and incubated overnight. The previously determined concentration of polybrene in complete medium with either α2β1 shRNA Lentiviral Particles, Control shRNA Lentiviral Particles-A, or no shRNA Lentiviral Particles (non-transduced) were added and incubated for 18 hours [multiplicity of infection (MOI) of 1]. The polybrene/lentivirus solutions were removed, and the cells were allowed to incubate in complete medium for 2 days. Stable clones were selected using the optimal concentration of puromycin dihydrochloride as determined previously until the non-transduced cells were killed. Puromycin dihydrochloride containing medium was replaced every 3–4 days and cell splitting was performed at any point when cells reached a confluency of 80%.

### Integrin α2β1 Knockdown Refinement.

To select highly KD cells, a modified adhesion assay was performed due to the high affinity of integrin α2β1 to collagen type I. A 6-well plate was coated at 1 μg/cm^2^ of collagen type I in sterilized 0.02M acetic acid in Milli-Q water overnight on a plate shaker in 4°C (1.5 mL/well). Three wells were needed per cell line. The next day, each well was washed three times in 1X PBS for 5 minutes on a plate shaker. Cells were detached from the T25 flask using 1X Trypsin EDTA and resuspended in 1.5 mL of complete medium after centrifugation. The cells were added to each well of the collagen-coated 6-well plate and allowed to sit for increasing time increments (10 minutes, 25 minutes, and 35 minutes) in the incubator. Floating cells were moved from one well to the next well since the cells with fewer α2β1 integrins will take longer to adhere. Once complete, the remaining floating cells were added to a new T25 flask and cultured until sufficient cells were present for further experimentation.

### 2D Fluorescent Staining.

Cells were seeded at 10,000 cells/well in a 96-well flat bottom plate. The following day, the wells were fixed for 25 minutes with 4% paraformaldehyde (PFA), blocked for 60 minutes, primary antibody was added for 90 minutes, followed by a primary-specific secondary antibody and a 1:1000 dilution of DAPI for 1 hour, all at 100 μL per well (Table 1). Between each addition, wells were washed with 150 μL of 1X PBS and all steps were done on a plate shaker. One well per condition did not have any primary antibody to serve as a secondary only control to ensure non-specific binding is absent. Goat anti-mouse IgG (H + L) highly cross-adsorbed secondary antibody Alexa Fluor plus 594 and goat anti-rabbit IgG (H + L) highly cross-adsorbed secondary antibody Alexa Fluor 488 were used as the secondary antibodies for mouse, and rabbit hosts, respectively. Wells were imaged using an Agilent BioTek Cytation 5 Cell Imaging Multimode Reader. The images were captured with a 10X, 0.3 NA objective in a 4x4 montage (3.6% overlap) with a 75% crop field of view to only image the inside of the wells (16-bit TIF, 1496 x 1496 pixels, 1043 x 1043 μm). The DAPI, GFP, and Texas Red filter cubes were used to capture the DAPI, Alexa Fluor 488, and 594 signals, respectively. Image analysis was completed using the built-in Gen5 Software. Cells were segmented using the DAPI channel using a threshold value of 5000, a minimum object size of 5 μm, and a maximum object size of 35 μm. The primary mask was expanded to include the entire cell by setting a threshold value above the background value with a maximum expansion radius of 50 μm. All holes were filled in both masks. Each detected cell was exported to a CSV file for analysis in RStudio. The median integrin intensity signal of each well was calculated and used for statistical analysis.

### Western Blotting Sample Preparation.

Cells were plated in a 100 mm tissue culture treated dish and grown until 80% confluency was achieved. Cells were detached using 1X Trypsin EDTA and then washed three times with ice cold 1X PBS, centrifuging at 250 rcf for 5 minutes between washes. Cells were counted using a hemocytometer and resuspended in Santa Cruz Biotechnology RIPA lysis buffer system with phosphatase inhibitor cocktail (1:100) and EDTA (1:100) at 1 x 10^7^ cells/mL of buffer. The samples were vortexed on high for 30 seconds and then sonicated three times in 30 second intervals using a VWR Ultrasonic Cleaner. The samples were placed on a shaker at high speed for 25 minutes at 4°C then centrifuged at 14,000 rcf for 15 minutes at 4°C. The supernatant was pipetted into a new 1.5 mL tube. A BCA Protein Assay Kit was used to determine protein concentration of the samples. BSA standards suspended in the same RIPA lysis buffer as the samples were created ranging from 0–2 mg/mL. 5 μL of sample or standard was added to 100 μL of working reagent in duplicate in a 96 well plate. The plate was placed on a shaker at a high speed for 30 seconds and then placed in an incubator at 37°C for 30 minutes. Absorbance was measured at 562 nm using an Agilent BioTek Cytation 5 Cell Imaging Multimode Reader. The average of each BSA standard was used to create a linear line of best fit to which the sample protein concentrations could be determined (typically ~ 1.5-2 mg/mL). The samples were stored at −20°C.

### Western Blotting.

The samples were slowly thawed on ice and Invitrogen NuPAGE Bis-Tris chemistry was used. Western blot samples were prepared using manufacturer protocols with 15 μg of protein in each 25 μL sample. The samples were heated at 70°C for 10 minutes before loading into a NuPAGE Bis-Tris 4–12% Mini Protein Gel with one lane loaded with 15 μL of protein ladder. 1X NuPAGE MOPS SDS running buffer with 0.25% NuPAGE antioxidant was added with the gel in an Invitrogen Mini Gel Tank. The gel was run at 60V through the stacking portion of the gel (~ 15 minutes) and then at 200V until the sample front was at the bottom of the gel (~ 45 minutes). A 0.45 μm nitrocellulose membrane, two filter papers, and two sponges were pre-soaked in 1X NuPAGE transfer buffer with 20% methanol and 0.1% NuPAGE antioxidant for 15 minutes prior to transfer. After electrophoresis, the gels were removed from the cassette, and the protein was transferred to the membrane in the Mini Gel Tank for 90 minutes at 20V in a Mini Blot Module. Ponceau S Staining Solution and Coomassie Brilliant Blue R-250 Staining Solution were used to visualize transferred protein in the membrane and residual protein left on the gel after transfer, respectively (per manufacturer protocols). After removing the Ponceau S Staining Solution from the membrane with 0.1% NaOH, it was blocked with 5% nonfat dry milk in 1X TBST (20 mM, 150 mM NaCl, 0.1% w/v Tween-20 detergent in Milli-Q water) for 60 minutes at room temperature on a plate shaker. The membrane was washed three times in 1X TBST for 5 minutes each wash and primary antibodies for integrin α2β1 (1:1000) and β-tubulin (1:5000; loading control) were added in 5% BSA in 1X TBST overnight at 4°C on a plate shaker. The next day, the membrane was washed three times in 1X TBST for 20 minutes each wash and then Goat anti-Mouse IgG (H + L) Secondary Antibody, HRP was added at 1:10,000 dilution in 5% BSA in 1X TBST to recognize both primary antibodies. The membrane was washed three times with 1X TBST for 20 minutes each wash and then developed first with SuperSignal West Pico PLUS Chemiluminescent Substrate for proper β-tubulin signal (loading control) and then again with SuperSignal West Atto Chemiluminescent Substrate for the integrin signal. Images were acquired with an iBright FL1500 Imaging System and analyzed automatically in the iBright Analysis Software (Version 5.4.0).

### Spheroid Slicing and Staining.

After the 72-hour migration experiment was complete, samples were washed three times with 150 μL of 1X PBS and fixed for 25 minutes with 100 μL of 4% PFA on a plate shaker. The samples were washed three times with 150 μL of 1X PBS and embedded in Scigen Tissue-Plus^™^ O.C.T. Compound. They were then sliced at 30 μm using a Cryotome FSE, placed on Permafrost Adhesion Slides, and allowed to dry at room temperature for 1 hour before storing at −80°C. Prior to staining, the slides were warmed up to room temperature and washed three times with 1X PBS. Ibidi Sticky-Slide Tissues were placed on the slides. Then the slides were blocked for 60 minutes, primary antibody was added for 90 minutes, followed by a primary-specific secondary antibody (Alexa Fluor Plus 594) and 1 μg/mL DAPI for 1 hour, all at 160 μL per slide (Table 1). Between each addition, the slides were washed with 150 μL of 1X PBS and all steps were done on a plate shaker. Samples were imaged using an Agilent BioTek Cytation 5 Cell Imaging Multimode Reader with a 10X, 0.3 NA objective (16-bit TIF, 1992 x 1992 pixels, 1389 x 1389 μm) using the Texas Red filter cube to capture the Alexa Fluor 594 signal and the DAPI filter cube to capture the nuclear staining.

### Adhesion Assays.

A non-treated 96 well plate was coated at 5 μg/mL of denatured (boiled in a water bath for 10 minutes) collagen type I in 1X PBS overnight on a plate shaker at 4°C. The plate was then washed three times with 1X PBS and blocked with 1% BSA at 37°C (BSA control wells were included by blocking non-collagen coated wells). Meanwhile, cells were detached using 1X Trypsin EDTA and then washed three times with 1X PBS. Cells were counted using a hemocytometer and resuspended at 600,000 cells/mL in adhesion buffer [88.8% DMEM/F12, 10% BSA (5% stock solution in 1X PBS), 1% magnesium chloride (100 mM stock solution in Milli-Q water), and 0.2% manganese chloride (100 mM stock solution in Milli-Q water)]. 100 μg/mL of either mAb D93, human IgG1 isotype control antibody, or a non-antibody control in adhesion buffer was added at 50 μL in triplicate 30 minutes before the cells were added to allow for proper antibody attachment to the collagen. The cells were then added at 50 μL per well and allowed to adhere to the collagen (7 minutes for M4 cells and 9 minutes for MDA-MB-231 cells). The floating cells were gently pipetted out of the wells and the wells were washed gently twice with 1X PBS. 100 μL of crystal violet (1:20 dilution in 1X PBS of a 100 mg crystal violet stock solution in 50 mL of methanol) was added to each well for 10 minutes before gently washing the wells twice with 1X PBS. The plate was allowed to dry. The dried crystal violet was destained using 10% acetic acid in Milli-Q water for 5 minutes on a shaker. The absorbance value was read at 600 nm using an Agilent BioTek Cytation 5 Cell Imaging Multimode Reader.

### D93 Staining.

After the 72-hour migration experiment was complete, samples were washed three times with 150 μL of 1X PBS and fixed for 25 minutes with 100 μL of 4% PFA on a plate shaker. The samples were washed three times with 150 μL of 1X PBS. 100 μL of 20 μg/mL goat-anti human IgG Alexa Fluor Plus 488 and 1 μg/mL DAPI in 2.5% bovine serum albumin (BSA) were added to the samples for 2 hours in the dark (Table 1). Samples were washed three times with 1X PBS and stored for imaging at 4°C.

### Two-Photon Microscopy.

Two-photon excitation and Second Harmonic Generation (SHG) imaging was completed on a custom-built microscope described here.^49^ The system is based on an uprigith Olympus BX50WI microscope base equipped with a Fluoview300 laser scanning unit (Olympus), a tunable Coherent Chameleon Ultra II Titanium laser, and a electro-optic modulator (ConOptics) for power modulation. Signals are detected using two H7421 GaAsP photomultiplier tubes (Hamamastsu) arranged in forward and backward detection geometries, with the forward detection path incorporating a 0.9 NA condenser lens. The collagen-embedded spheroid models that underwent the 72-hour migration experiment and D93 staining were carefully transferred to a well in a 3D printed holder (made in SOLIDWORKS Student Edition 2024 SP5.0) that was glued to a glass slide with Loctite 401 adhesive (**Supplementary Fig. 8**). Then, the wells were filled with tris-glycerol mounting solution (10% 0.1M tris, pH 9.0 in cell culture grade glycerol), a coverslip was placed on top, and the samples were allowed to equilibrate for 1 hour prior to imaging. Imaging was performed using a LUMPlanFLN 40X, 0.8 NA water emersion objective (Olympus) with 2X optical zoom, yielding a digital resolution of 512 x 512 pixels and using a laser scanning speed of 2.71 s/frame. At each location, two 100 μm z-stacks were acquired with a step size of 1μm. For SHG imaging, samples were excited at 890 nm, and SHG signals were collected using 448/20 nm bandpass filters in the forward and backward channels. For fluorescence imaging, an excitation wavelength of 780 nm was used with a 448/20 nm (Semrock) bandpass filter in the backward channel to collect the DAPI signal and a 535/50 nm (Semrock) bandpass filter in the forward channel to collect mAb D93 signal. The laser was operated in a power range of 7 to 12 mW for SHG imaging and 15 to 27 mW for fluorescence imaging at the focal plane. Six z-stack locations were captured per spheroid model: two next to the spheroid boundary (sphere region), two at the migrating cell front (cells region), and two at distant, non-cellular (far regions) sites.

### Image Analysis: F/B Ratio.

In FIJI, the forward and backward SHG image stacks were imported, and the forward stack was divided by the backward stack using the image calculator set to create a 32-bit stack. A mask was generated of the forward SHG image stack with the Li threshold method using the stack histogram. The median F/B Ratio per stack was measured only within the mask. The CSV files generated were saved and imported to RStudio for statistical analysis.

### Image Analysis: MAb D93 Intensity.

In FIJI, the median intensity of each mAb D93 image was calculated and saved to a CSV file. In RStudio, the median intensity of the entire stack was calculated and used in statistical analysis.

### Image Analysis: Anisotropy.

The 2D wavelet transform modulus maxima (WTMM) anisotropy method was implemented as previously described using Python (Version 3.13).^36,37^ For images in the far and sphere regions of the model, 15 forward SHG images for each z-stack (closest to the midpoint of the spheroid) were max projected every 3 slices. For images in the cells region, the cell nuclei were automatically segmented using the DAPI images in FIJI by smoothing and thresholding with the Otsu method using the stack histogram. Then the forward SHG image z-stacks were max projected every 3 slices to form 33 forward SHG images per z-stack (the last image in the 100-image z-stack was disregarded). The 2D WTMM was run on the max projected images of all regions by weighing the argument of local maxima by their modulus and generating probability density functions from all local maxima which the anisotropy was calculated at each wavelet scale. For the cells region, a 50-pixel radius circle was kept around each individual cell, excluding the DAPI segmented area. If a group of cells was detected (radius larger than 100-pixels along the long axis), the long axis was split evenly into three segments for three individual 50-pixel radius circles to be kept, excluding the DAPI segmented area. The analysis in all regions was completed over 27 wavelet size scales for reliable statistics.

### Image Analysis: CT-FIRE.

The CT-FIRE algorithm (version 3.0) was used on forward SHG images (max projected by 3) to explore individual fiber characteristics (angle, straightness, length, and width) in greater detail.^38^ Images were run as a batch with the following parameters: minimum fiber length of 10 pixels, image resolution of 3 dpi, fiber line width of 0.5, maximum fiber width of 15 pixels, and 10 histogram bins. The histograms from each image were imported into RStudio. The median of each fiber characteristic per z-stack was calculated and plotted as one point.

### Statistics.

The Shapiro-Wilk test was used to confirm if the set of data came from a normally distributed population while the Levene test was used to assess variances between groups. A Student’s t-test (2 groups) or ANOVA (3 + groups) was used if all groups were parametric and had equal variances. A Welch’s t-test (2 groups) or Welch’s ANOVA (3 + groups) was used if all groups were parametric but had unequal variances. Lastly, a Wilcoxon rank sum test (2 groups) or Kruskal-Wallis test (3 + groups) was used for nonparametric data regardless of variances. Either a Tukey HSD (post ANOVA), a Games-Howell (post Welch’s ANOVA), or a Dunn test (post Kruskal-Wallis) was used to identify which group average was statistically significant from one another when 3 + groups were compared. In these cases, significance is indicated on figures by letters where groups that do not share the same letter are significantly different.

## Figures and Tables

**Table 1:** Specific fluorescent staining parameters for each target. NA = not applicable. NFDM = nonfat dry milk. BSA = bovine serum albumin. PBS = phosphate buffered saline. LED Int = LED intensity. Int Time = integration time (in milliseconds). Cam Gain = camera gain.

Target	Host Species of Primary Antibody	Blocking Solution (in 1X PBS)	Primary Antibody (in 1X PBS)	Secondary Antibody (in 1X PBS)	Cytation 5 Exposure Settings
D93	Humanized Mouse	NA	20 μg/mL in 2.5% BSA	1:100 in 2.5% BSA	NA
Integrin α1β1	Mouse	5% NFDM	1:1000 in 5% BSA	1:400 in 5% BSA	LED Int: 10 Int Time: 1100 Cam Gain: 24
Integrin α2β1	Mouse	5% NFDM	1:500 in 5% BSA	1:400 in 5% BSA	LED Int: 10 Int Time: 350 Cam Gain: 24
Integrin α10β1	Rabbit	5% NFDM	1:200 in 5% BSA	1:400 in 5% BSA	LED Int: 10 Int Time: 81 Cam Gain: 24
Integrin α11β1	Mouse	5% NFDM	1:250 in 5% BSA	1:400 in 5% BSA	LED Int: 10 Int Time: 164 Cam Gain: 24
Integrin αVβ1	Rabbit	5% NFDM	1:200 in 5% BSA	1:400 in 5% BSA	LED Int: 10 Int Time: 27 Cam Gain: 24
DAPI	NA	NA	NA	1:1000	LED Int: 10 Int Time: 5 Cam Gain: 10

## Data Availability

All data and code supporting the findings of this study are available from the corresponding author upon reasonable request.
